# Impact of a Quality Improvement Initiative to Reduce Medication-induced Kidney Injury

**DOI:** 10.1097/pq9.0000000000000912

**Published:** 2026-09-04

**Authors:** William Stoudemire, Nour Akil, Adam Bernstein, Erica Bjornstad, Meredith Cuthriell, Nicole D. Damari, Darren Dewalt, Carmen Echols, Christopher Falato, Yuting Liao, Ceila Loughlin, Cameron McKinzie, Michael Steiner, Katie Westreich

**Affiliations:** From the * Department of Pediatrics, University of North Carolina at Chapel Hill School of Medicine, Chapel Hill, N.C.; † Department of Pediatrics, Duke University School of Medicine, Durham, N.C.; ‡ Department of Pharmacy, University of North Carolina Hospital, Chapel Hill, N.C.; § Department of Pediatrics, University of Tennessee Health Science Center College of Medicine, Memphis, Tenn.; ¶ UNC Health, Chapel Hill, N.C.; ‖ Department of Pediatrics, University of Cincinnati College of Medicine, Cincinnati, Ohio; ** Department of Pharmacy, Children’s Hospital Los Angeles, Los Angeles, Calif.

## Abstract

**Introduction:**

Acute kidney injury (AKI) is a common complication of nephrotoxic medication use in hospitalized children. Prior studies have shown that the use of electronic health record alerts to identify patients exposed to nephrotoxic medications reduced exposure and the incidence of AKI. This initiative aimed to reduce the incidence of AKI by implementing a process to identify nephrotoxic exposures and standardize care for patients receiving nephrotoxic medications.

**Methods:**

We used quality improvement methodology to identify and reduce nephrotoxic medication exposure and to standardize hydration and monitoring for patients receiving them. We tested the process on a single hospital service before expanding to other services, with ongoing optimization via monthly Plan-Do-Study-Act cycles.

**Results:**

During the 48-month study period, we observed a significant decrease in the mean number of patients exposed to nephrotoxic medications, from a baseline of 11.3 patients per 1,000 patient-days per month to 6.6 patients per 1,000 patient-days per month, a 42% decrease. For patients exposed to nephrotoxic medications, there was an increase in AKI monitoring (via increased creatinine testing) and an increase in the percentage who developed AKI.

**Conclusions:**

We adapted and implemented a screening process for exposure to nephrotoxic medications, along with a novel hydration and monitoring pathway. Consistent with previous studies, we observed a dramatic decrease in exposure. An increase in the detection of AKI and a reduction in the proportion of lower risk patients may explain the unexpected increase in the percentage who developed AKI. Our study suggests that implementing standardized identification and monitoring processes can reduce exposure, and that automation and adherence monitoring are important for demonstrating reductions in AKI.

## INTRODUCTION

Acute kidney injury (AKI) is common during hospitalization. A substantial proportion of AKI in hospitalized patients is due to iatrogenic injury from nephrotoxic medications.^1,2^ Exposure to these agents occurs in up to 86% of noncritically ill hospitalized pediatric patients.^3^ Although there is active research on strategies to improve outcomes for patients with AKI, none has been highly successful, emphasizing the need to prevent AKI.^4^

### Our Problem

Before this initiative, there was no standardized way of preventing or monitoring kidney injury caused by nephrotoxic medication exposure. An incident of iatrogenic medication-associated AKI severe enough to necessitate dialysis and a 3-month hospital stay reinforced the gravity of this problem.

### Available Knowledge and Rationale

AKI causes both short- and long-term morbidity. AKI includes 3 stages: stage 1 (serum creatinine 1.5–1.9 times baseline or ≥ 0.3 mg/dL increase), stage 2 (2.0–2.9 times baseline), and stage 3 (≥3 times baseline, creatinine ≥ 4.0 mg/dL, or initiation of dialysis).^5^ Hospitalized patients experiencing AKI, including those with stage 1 AKI, have increased length of stay and mortality risk.^6,7^ Evidence also suggests long-term consequences: pediatric patients with nephrotoxic medication-associated AKI may have residual renal dysfunction months later, and recurrent AKI episodes increase the risk of long-term kidney dysfunction.^2,8^

AKI resulting from nephrotoxic medications is frequently underrecognized yet often preventable.^9^ The NINJA (Nephrotoxic Injury Negated by Just-in-time Action) project, developed at Cincinnati Children’s Hospital, reduced exposure to nephrotoxic medications and the rates of medication-associated kidney injury.^10–12^ Interventions included using software to identify patients receiving nephrotoxic medications, triggering increased monitoring and hydration recommendations. As a result, the project achieved a 38% decrease in exposure to nephrotoxic medications, a 40% reduction in the rate of AKI among patients exposed to nephrotoxic medication, and a 64% reduction in the hospital-wide incidence of AKI. The project sustained these improvements for 4 years and proved translatable to other hospitals.^12,13^

### Specific Aims

We aimed to reduce 2 metrics: (1) exposure to nephrotoxic medications, which we defined as the use of 1 intravenous aminoglycoside or 3 or more additional nephrotoxic medications, and (2) AKI resulting from nephrotoxic exposure.

To establish our baseline, we reviewed 18 months of data from patients exposed to nephrotoxic medications at the children’s hospital, excluding the neonatal critical care unit and hematology/oncology services due to their unique patient populations and management strategies. The average number of exposed patients was 11.3 per 1,000 patient-days, and 23.0% of exposed patients developed AKI. Based on those findings, we established the following specific aims:

1. Reduce the number of patients exposed to nephrotoxic medications by 25% within 1 year.
2. Reduce the percentage of patients exposed to nephrotoxic medications who develop AKI by 25% within 1 year.

## METHODS

### Context

We implemented this initiative at a medium-sized, suburban quaternary academic children’s hospital with 150 inpatient beds and 20 intensive care unit beds. Approximately 6,500 children are admitted each year. The children’s hospital is located within a larger, public university–affiliated medical center and serves the southeastern United States. Patients are cared for by a variety of general hospitalists and subspecialty services. Approximately 60% of patients receive Medicaid, and 25% have private insurance. The hospital uses the Epic Systems (Verona, WI) electronic health record (EHR).

### Improvement Process and Interventions

Seeing an opportunity to build on the success at other institutions, we formed a multidisciplinary improvement team, including attending and fellow physicians from pediatric pulmonology and pediatric nephrology, pharmacists, nurse coordinators, floor nurses, and a medical student. Two executive sponsors—the director of inpatient hospital services and the director of pediatric pharmacy services—provided support and oversight.

**Fig. 1.**
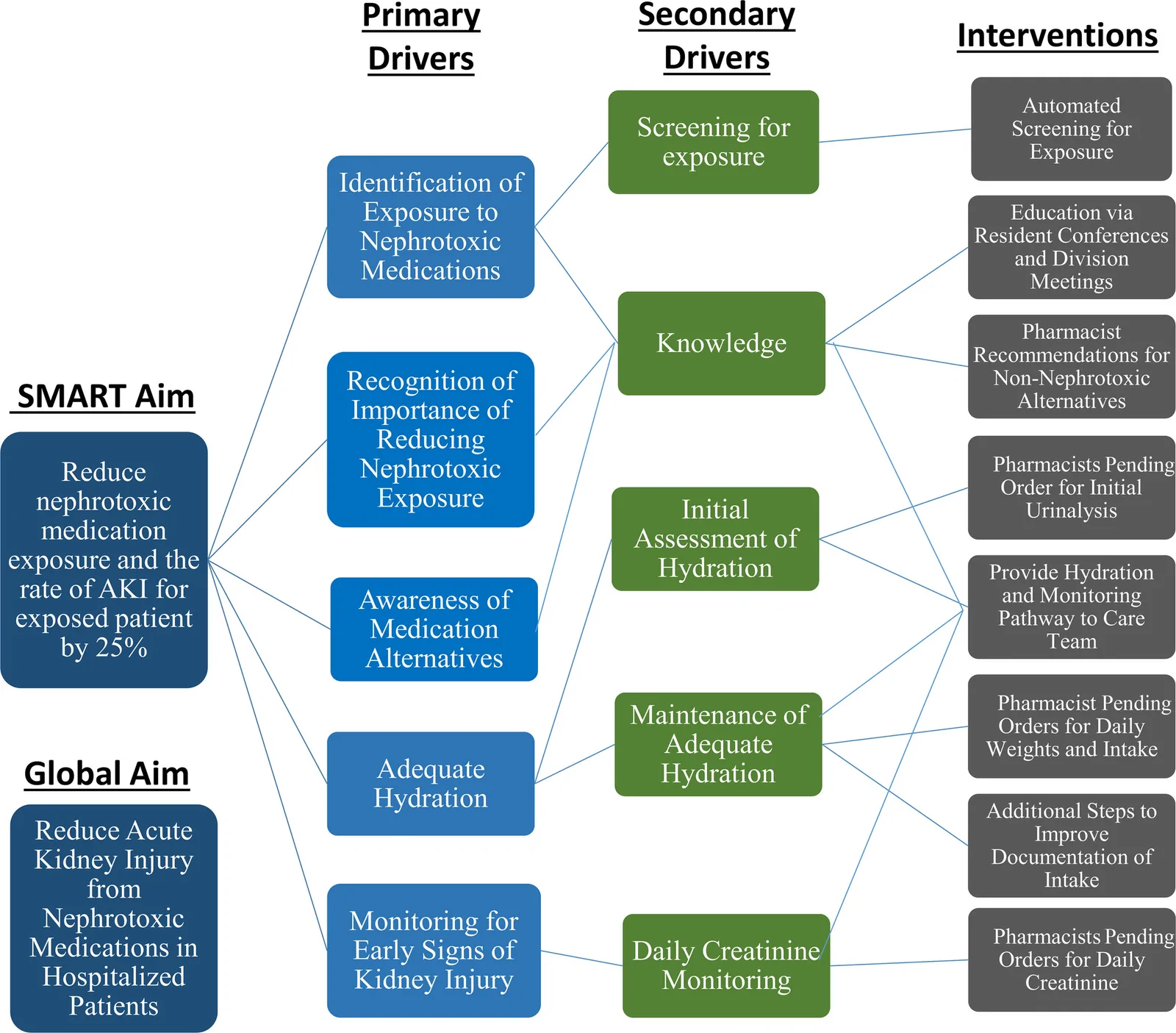
Key driver diagram.

**Fig. 2.**
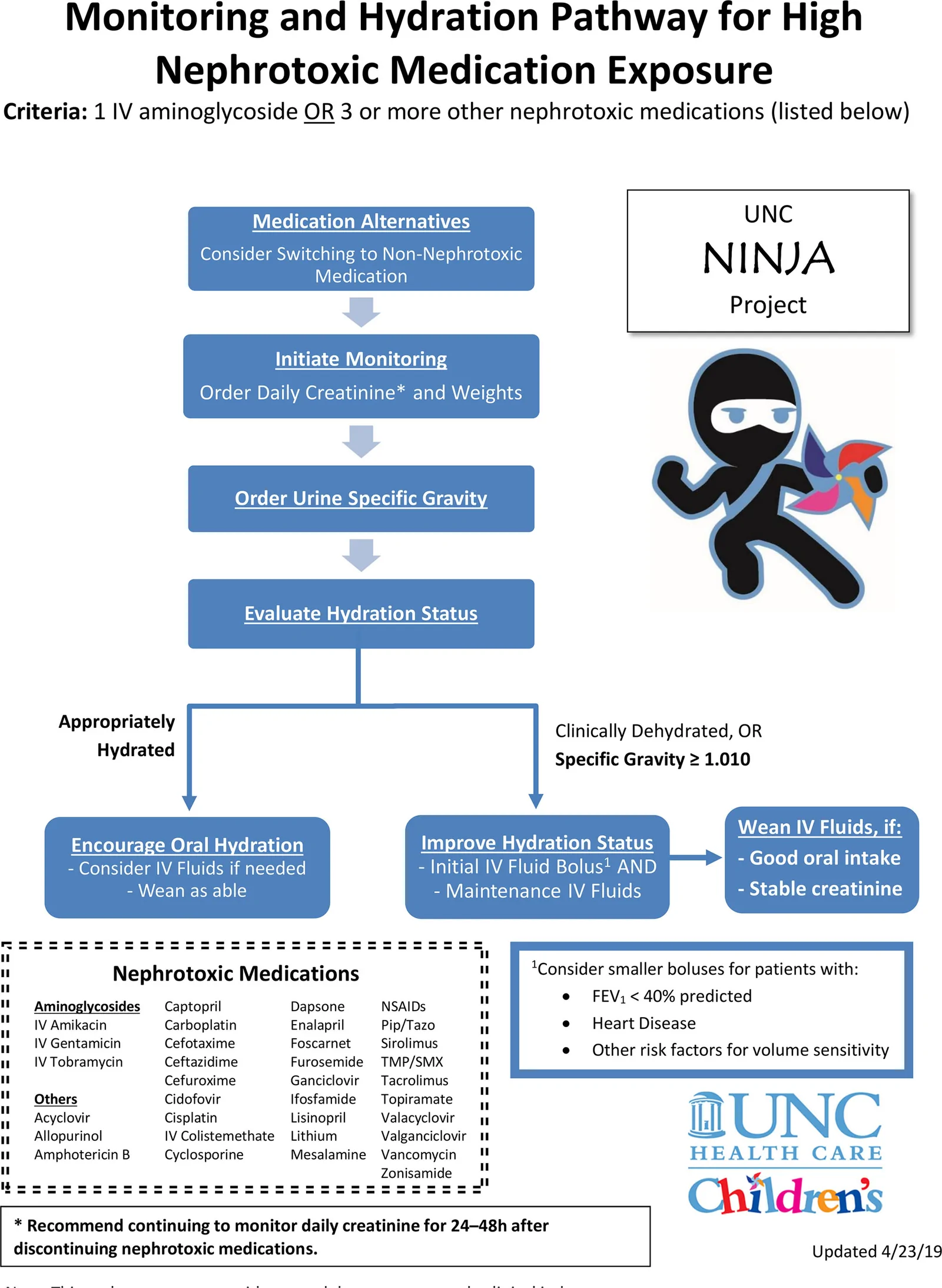
The monitoring and hydration pathway, including medications considered nephrotoxic. IV, intravenous; NSAIDs, non-steroidal anti-inflammatory drugs; TMP/SMX, trimethoprim/sulfamethoxazole.

**Fig. 3.**
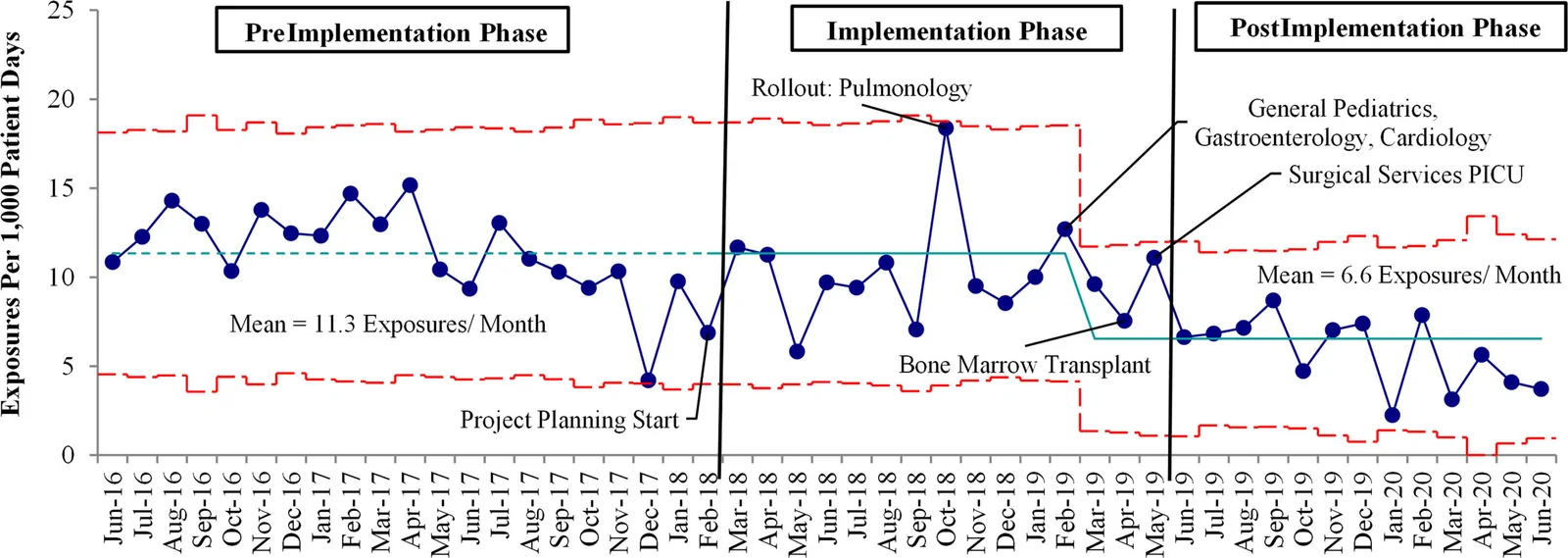
Statistical process control U-chart for the mean number of patients exposed to nephrotoxic medications per 1,000 patient-days. Red dashed lines represent the upper and lower control limits. The center line is green and represents the mean.

We used quality improvement tools, including a key driver diagram (Fig. 1), Pareto analysis, root cause diagram, and process mapping, to plan interventions. (**See figure 1, Supplemental Digital Content 1**, which displays the Pareto analysis of the primary factor [as determined by a reviewer performing chart review] for 20 patients who developed AKI while exposed to nephrotoxic medications, https://links.lww.com/PQ9/A804.) (**See figure 2, Supplemental Digital Content 2**, which displays the root cause analysis of different factors contributing to the development of medication-induced kidney injury, https://links.lww.com/PQ9/A805.) (**See figure 3, Supplemental Digital Content 3**, which displays the process map of the proposed process for screening patients for exposure to nephrotoxic medications and enacting the management pathway, https://links.lww.com/PQ9/A806.) Stakeholder collaboration determined feasibility and identified operational targets for improvement. Pareto analysis identified services with the highest exposure burden and AKI incidence.

Key drivers included identifying patients exposed to highly nephrotoxic medications, suggesting non-nephrotoxic alternatives, ensuring adequate hydration before and during medication administration, and monitoring for early kidney injury. We developed a screening method using daily EHR reports to identify patients receiving predefined nephrotoxic medications. The team’s pharmacist reviewed these reports daily, and we refined them over multiple Plan-Do-Study-Act cycles to improve accuracy in identification. We also created a standardized care pathway with recommendations for alternative medications, hydration, and monitoring for exposed patients. This pathway was adapted from those used at other institutions to fit our local hospital system while allowing individualization based on patient needs (Fig. 2).

We implemented the screening process and monitoring/hydration pathway simultaneously on the pediatric pulmonology service and refined them through monthly Plan-Do-Study-Act cycles. After qualitative and quantitative data indicated success, we expanded the interventions to other medical and surgical services, with continued monthly refinement and staff education.

### Measures

#### Process Measure

We used the number of serum creatinine levels drawn per encounter as a process measure to assess pathway uptake.

#### Primary Outcome Measure

The mean number of patients exposed to nephrotoxic medications per 1,000 patient-days (a secondary driver based on hospital census data) per month is shown in Figure 3. We defined exposure as a patient receiving nephrotoxic medications during an individual hospital encounter. Patients were counted only once if they received nephrotoxic medications more than once during a hospitalization or were exposed for more than 1 month. Exposure and AKI were attributed to the month during which the nephrotoxic antibiotics were first initiated during the hospitalization.

#### Secondary Outcome Measure

The mean monthly AKI rate among exposed patients. We defined AKI as a serum creatinine value during the nephrotoxic exposure period that was more than 1.5 times the baseline serum creatinine or more than 0.3 mg/dL above the lowest serum creatinine value in the prior 6 months, or, if no prior creatinine was available, the first serum creatinine measured during the hospitalization.

#### Balancing Measures

1. Number of central line infections (given increased laboratory draws from central lines, which were highly prevalent in our pilot population).
2. Physician diagnosis of fluid overload given increased hydration (based on a search of diagnostic codes associated with fluid overload for exposed patients).

### Study of the Interventions and Analysis

We studied the outcomes of our project using a time-series design, with preimplementation data collected 21 months before the active interventions began.

We used statistical process control charts to monitor changes in outcome measures, with control limits set at 3 SDs; a shift of 8 or more points above or below the mean indicated special cause variation. We collected qualitative data during Plan-Do-Study-Act cycles and the rollout to sequential services and used it to assess results and identify changes to optimize our interventions. We performed statistical analysis using Microsoft Excel and QI Macros (version 2022; KnowWare International, Inc.).

The institutional review board approved this project (institutional review board no. 18-1363).

## RESULTS

The study period began on June 1, 2016, with preimplementation data collected through February 2018. Data collection continued throughout implementation and for 13 months after full implementation, through June 30, 2020. We initially implemented the pathways on the pediatric pulmonology service, then expanded them to pediatric cardiology, pediatric gastroenterology, the 2 hospital pediatric services, and the remainder of the children’s hospital, excluding pediatric hematology/oncology and neonatal critical care.

### Process Measure

Uptake of the screening and hydration/monitoring pathways was robust. The frequency of serum creatinine monitoring increased across all phases. During the preimplementation period, there were 770.8 creatinine measurements per 1,000 days of nephrotoxic exposure (corresponding to monitoring on 77% of exposure days). This increased to 1,013.6 measurements per 1,000 exposure days (101%) during the implementation period and remained high in the postimplementation phase at 959.9 measurements per 1,000 exposure days (96%).

### Primary Outcome Measure

The rate of patients exposed to nephrotoxic medications decreased during the study period. During the preimplementation phase, the mean number of patients exposed to nephrotoxic medications was 11.3 per 1,000 patient-days per month. Statistical process control analysis showed a statistically significant process change beginning in February 2019, with a decrease in the mean number of exposures to 6.6 per 1,000 patient-days per month (Fig. 3). This decrease represented a 42% reduction in the mean number of patients exposed to nephrotoxic medications per month compared with baseline.

### Secondary Outcome Measure

**Fig. 4.**
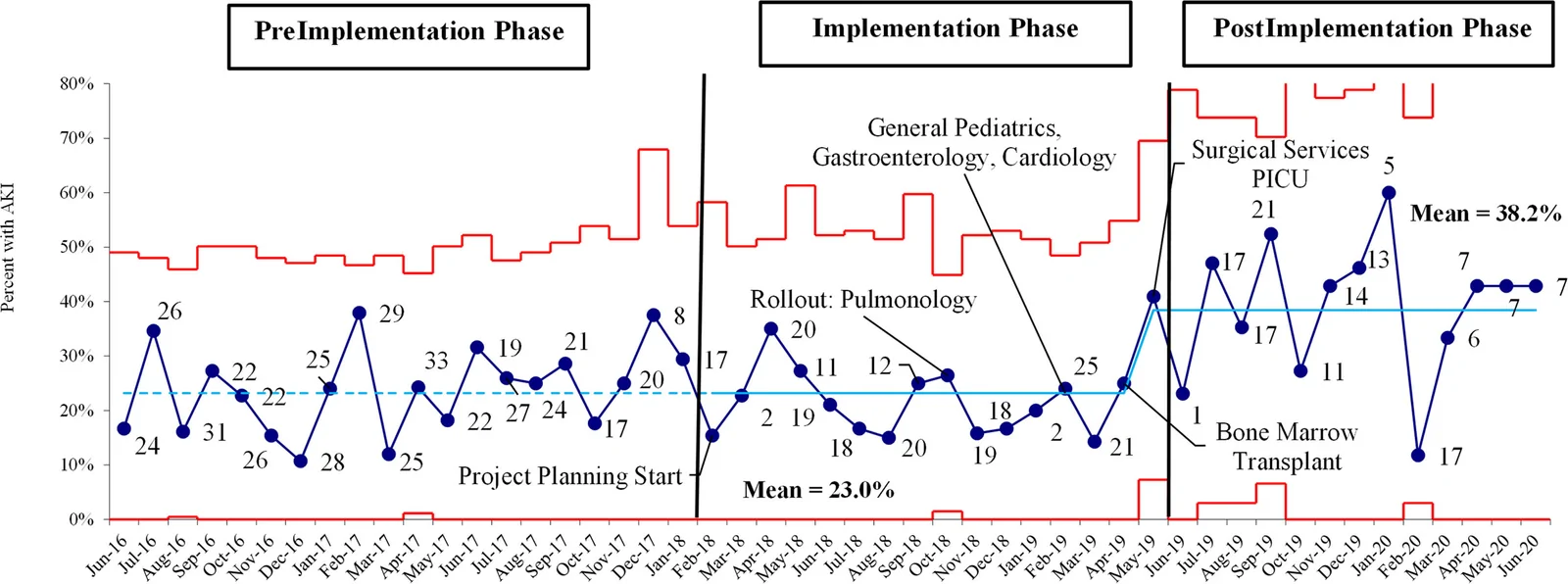
Statistical process control p-chart for the monthly percentage of AKI among patients exposed to nephrotoxic medications. Red dashed lines represent the upper and lower control limits. The center line is blue and represents the mean. The numbers above represent the absolute number of patients exposed to nephrotoxic medications per month.

**Fig. 5.**
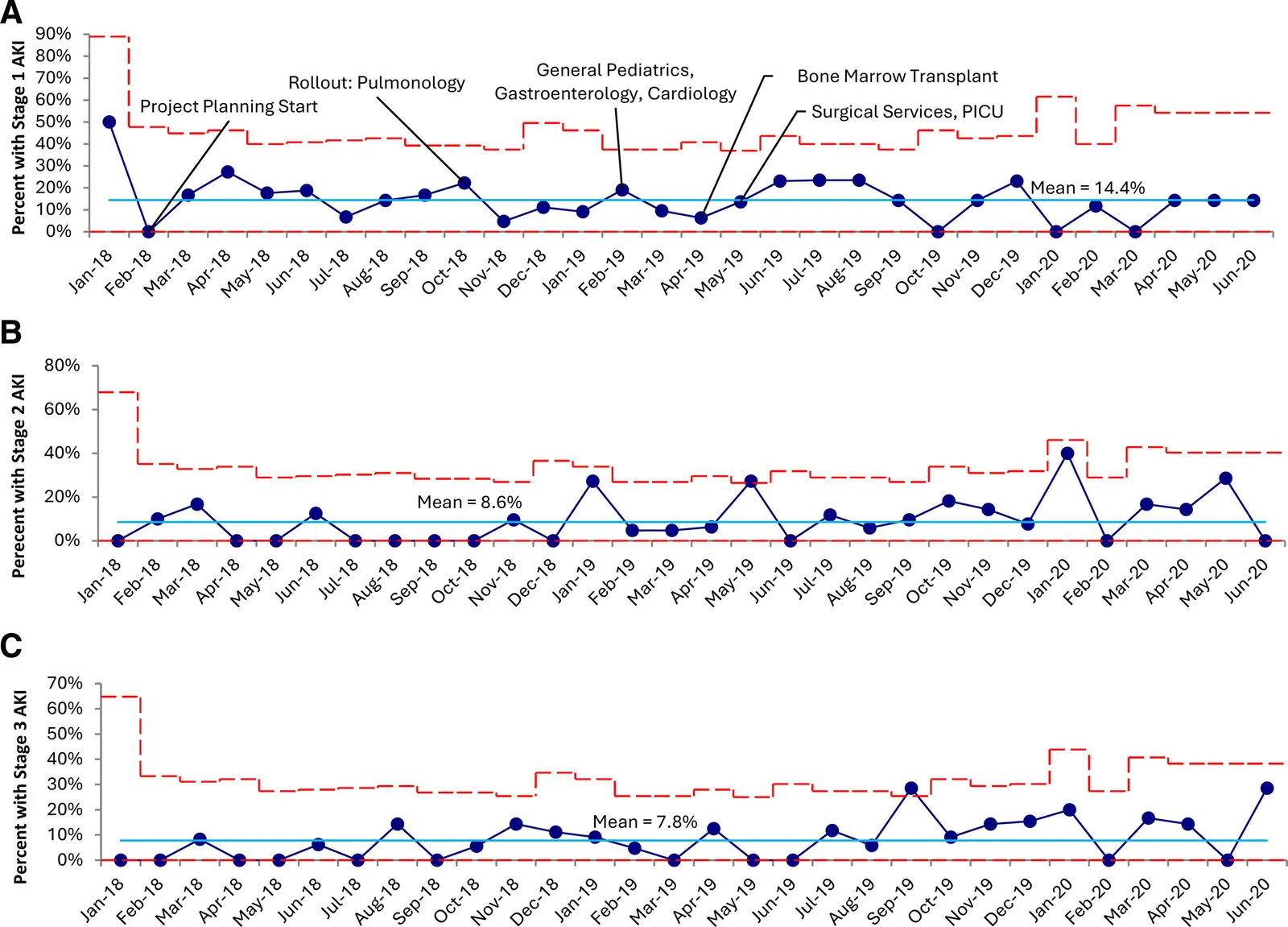
Statistical process control p-charts for the mean rates of stage 1 (A), stage 2 (B), and stage 3 (C) AKI among patients exposed to nephrotoxic medications. Upper and lower confidence limits are represented as red dashed lines. The blue center lines represent the means.

**Fig. 6.**
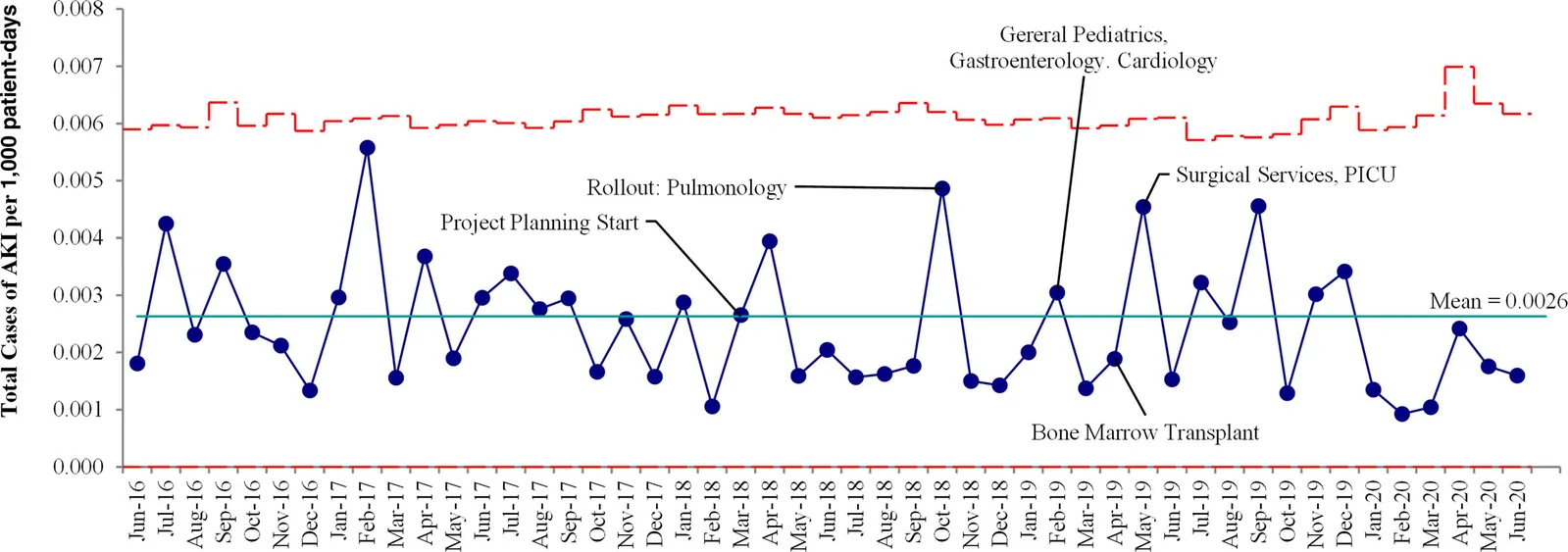
Statistical process control p-chart of AKI cases per 1,000 patient-days. Red dashed lines represent the upper and lower control limits. The center line is green and represents the mean.

The percentage of exposed patients who developed AKI per month was 23.0% during the preimplementation phase. Statistical process control analysis showed an increase to 38.2% (Fig. 4). When we analyzed the data across AKI stages, there were no significant differences in the distribution of AKI among stages compared with baseline (Fig. 5). Additionally, although there was a trend toward lower overall AKI cases per 1,000 patient-days, with 6 consecutive data points below the center line during the last 6 months of data collection, this decrease did not yet reach statistical significance (Fig. 6).

### Balancing Measures

We observed no cases of central line infections or episodes of fluid overload in patients included in the interventions.

## DISCUSSION

### Summary

Our team successfully implemented and optimized a clinical pathway to identify, hydrate, monitor, and offer alternatives to hospitalized children exposed to nephrotoxic medications. Adoption of the pathway was robust, evidenced by an increase in the mean number of serum creatinine measurements per encounter among exposed patients during the study period. We observed a dramatic decrease in the number of patients exposed to nephrotoxic medications during the study period: a 42% reduction in the mean monthly number of exposures compared with the preimplementation phase.

### Interpretation

The decrease in nephrotoxic medication exposure was consistent with Goldstein et al,^12^ who reported a 38% decrease after implementing automated EHR alerts, recommendations for non-nephrotoxic alternatives, and daily creatinine monitoring. Our process identified patients only after nephrotoxic medications had been ordered and given. Our theory is that repeated reminders with suggestions for non-nephrotoxic medication alternatives led to practice changes among care providers, such that the whole population benefited from more frequent use of non-nephrotoxic alternatives, a potential “halo effect.” Although automated EHR-embedded alerts may be more effective than manual review of daily reports, the automated EHR reporting mechanism developed by the NINJA group required significantly more resources and infrastructure than were available to us.^11^

Notably, improvement in the exposure rate seems to begin during the preimplementation period, with a potential centerline shift before formal rollout. This pattern may reflect a Hawthorne effect, in which increased awareness of the project and anticipated changes in practice influenced clinician behavior before full implementation. Our data suggest that increased awareness can significantly reduce exposure to these medications and may even occur to some degree before structured interventions. Therefore, less resource-intensive interventions, such as our project, may be more easily replicated in smaller hospitals without extensive resources.

In contrast to the NINJA group, which reported a decrease in AKI among exposed patients from 25.5% to 14.5%, we observed an increase.^12^ This may reflect increased creatinine monitoring and detection among patients who remained exposed. Patients who continued receiving nephrotoxic medications may also have been at higher risk because lower risk exposures were discontinued, whereas those requiring continued nephrotoxic therapy may have had greater severity of illness or other AKI risk factors. Finally, unlike NINJA, we included all patients receiving intravenous aminoglycosides rather than only those receiving them for 3 or more days. This more inclusive definition of exposure may have changed the risk profile of our exposed population and contributed to differences in the AKI percentage.

Differences in workflows may have also contributed to discordant findings between our project and the NINJA project. In our project, an EHR-generated report identified inpatients who met our nephrotoxin exposure definition each morning, and it was routed to the centralized pediatric pharmacy service and service-embedded pharmacists. Pharmacists aimed to act within 24 hours by suggesting non-nephrotoxic alternatives, pending daily serum creatinine orders during exposure, and reinforcing hydration per pathway. Communication occurred via EHR messages/pended orders and, when present, during bedside rounds. Unlike the NINJA model’s real-time, order-triggered alerts and centralized daily adherence auditing, our process required manual daily review. It did not mandate a response time or conduct a uniform daily performance audit. These factors, together with increased creatinine monitoring, may partially explain why our percentage of exposed patients who developed AKI did not decrease despite a substantial reduction in exposure.

Increased AKI detection may explain our findings. The baseline AKI percentage in our cohort (23.0%) was similar to that in Goldstein et al^10^ (25.5%), suggesting comparable underlying injury rates. The increase in AKI observed in our study may therefore partly reflect improved detection of previously unrecognized events. However, similar increases in monitoring did not preclude reductions in AKI rates in NINJA cohorts. This difference may reflect lower baseline monitoring at our institution, the inclusion of higher risk populations such as critically ill and surgical patients later in the study, and variability in the timing and reliability of interventions after exposure identification. These factors may have limited our ability to reduce true kidney injury.

The finding that our interventions reduced the number of children exposed to nephrotoxic medications but did not decrease the percentage of exposed patients who developed AKI or the overall number of AKI cases among exposed patients suggests that the identification of patients receiving nephrotoxic medications may have the highest yield. Alternatively, the monitoring and hydration components may have had less impact. There was not a shift from more severe AKI to less severe AKI, suggesting that increased hydration and monitoring for exposed patients may not reduce the likelihood of developing AKI or its severity.

Finally, the monitoring and hydration pathway may not have been adopted as consistently as intended. Although the pathway was well received and creatinine monitoring increased, we could not definitively assess whether patients received the recommended hydration and monitoring interventions. Reliance on a broad team response to pharmacist notifications may also have led to diffuse responsibility; a more centralized pharmacist role may have improved adherence.

Although we completed this project before the publication of quality improvement goals and consensus-based recommendations for pediatric AKI in 2021 and 2022, our approach was largely consistent with those recommendations.^14,15^ The authors recommended identifying high-risk situations for developing AKI, including exposure to nephrotoxic medications, considering alternative medications, and monitoring for the development of AKI, all of which our interventions incorporated. Our findings reinforce these recommendations and provide further evidence that such efforts can reduce exposure to nephrotoxic medications in hospitalized children.

## LIMITATIONS

Several limitations should be noted. We could not fully quantify pathway use, particularly whether patients received the recommended hydration and monitoring interventions. Our manual exposure identification process may have limited adoption compared with automated EHR alerts and centralized adherence auditing. Unmeasured factors during the study period, including patient type, indications for hospitalization, and changes in service mix, could explain the exposure and AKI trends. We also could not assess hospital-wide AKI rates or total nephrotoxic exposure days, which may better reflect overall exposure and would have allowed comparison with prior studies.

Patient populations may also have contributed to differences between our findings and prior efforts. We excluded the neonatal critical care unit because of distinct renal physiology, fluid management needs, and workflows, and we excluded hematology/oncology because many patients already receive protocolized hydration and frequent laboratory monitoring.^16^ Although the original NINJA project also excluded neonates, a subsequent neonatal initiative reduced nephrotoxic exposure and medication-induced AKI.^17^ Excluding these groups may have limited our ability to demonstrate benefits in populations where standardized practices could amplify intervention effects. Conversely, the later addition of intensive care and bone marrow transplant services may have increased the proportion of higher risk patients and influenced AKI trends.

## CONCLUSIONS

In conclusion, our hospital implemented a clinical pathway to identify, hydrate, and monitor hospitalized children, resulting in fewer patients being exposed to nephrotoxic medications. Our findings confirm those of prior studies: standardized processes for identifying patients exposed to nephrotoxic medications can reduce such exposure. However, these efforts did not produce a measurable decrease in the percentage of exposed patients who developed AKI or in the total number of AKI cases. Institutions aiming to reduce medication-induced AKI should consider real-time EHR exposure alerts, mechanisms to monitor adherence to management pathways, and automation whenever possible. Future studies should identify specific interventions that reduce AKI among children exposed to nephrotoxic medications.

## ACKNOWLEDGMENT

The authors wish to acknowledge the leadership at UNC Children’s Hospital for their support of this project.

## Supplementary Material


