# Supplementary material for: Impact of a Quality Improvement Initiative to Reduce Medication-induced Kidney Injury
Source: Pediatr Qual Saf. 2026 Sep 4;11(5):e912. doi: 10.1097/pq9.0000000000000912 (PMC13544836; doi:10.1097/pq9.0000000000000912)
Supplement: Supplementary file 1 [file pqs-11-e912-s001.pdf]

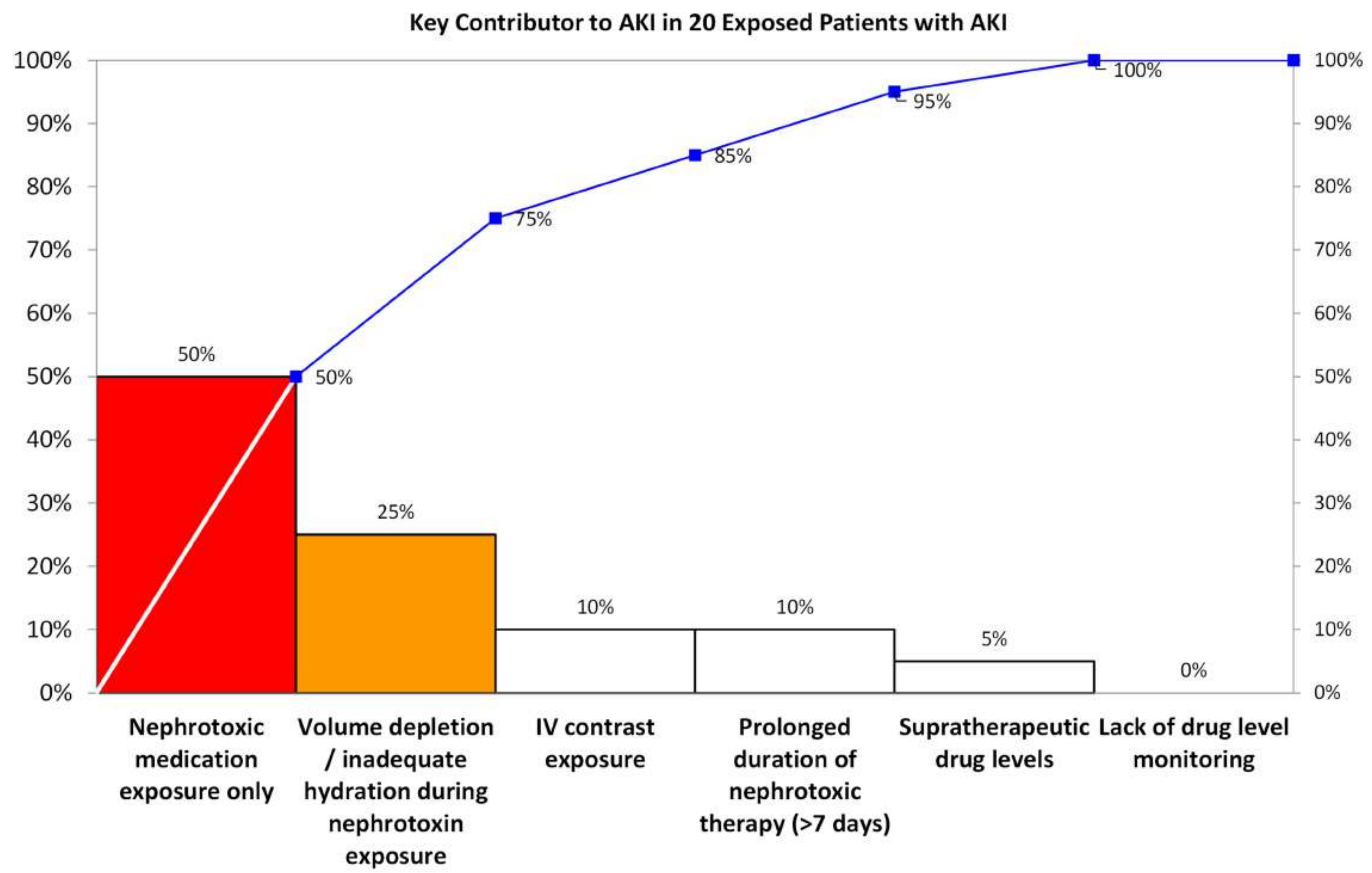

Key Contributor to AKI in 20 Exposed Patients with AKI
100%
90%
80%
70%
60%
50%
40%
30%
20%
10%
0%
50%
25%
10%
10%
5%
0%
50%
75%
85%
95%
100%
Nephrotoxic medication exposure only
Volume depletion / inadequate hydration during nephrotoxin exposure
IV contrast exposure
Prolonged duration of nephrotoxic therapy (>7 days)
Supratherapeutic drug levels
Lack of drug level monitoring
