# Supplementary material for: Impact of a Quality Improvement Initiative to Reduce Medication-induced Kidney Injury
Source: Pediatr Qual Saf. 2026 Sep 4;11(5):e912. doi: 10.1097/pq9.0000000000000912 (PMC13544836; doi:10.1097/pq9.0000000000000912)
Supplement: Supplementary file 2 [file pqs-11-e912-s002.pdf]

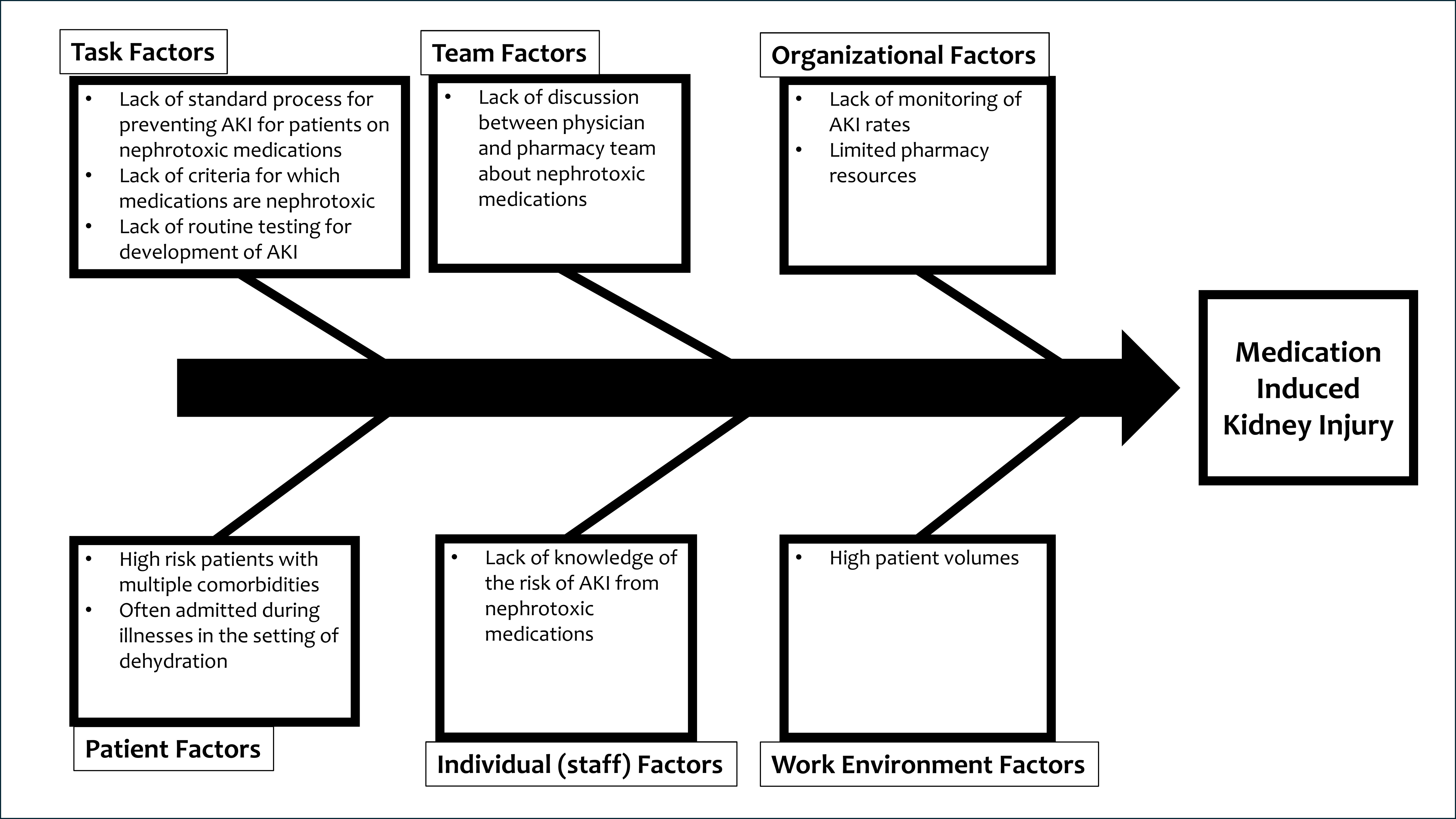

Task Factors
Lack of standard process for preventing AKI for patients on nephrotoxic medications
Lack of criteria for which medications are nephrotoxic
Lack of routine testing for development of AKI
Team Factors
Lack of discussion between physician and pharmacy team about nephrotoxic medications
Organizational Factors
Lack of monitoring of AKI rates
Limited pharmacy resources
Medication Induced Kidney Injury
High risk patients with multiple comorbidities
Often admitted during illnesses in the setting of dehydration
Patient Factors
Lack of knowledge of the risk of AKI from nephrotoxic medications
Individual (staff) Factors
High patient volumes
Work Environment Factors
