# Supplementary material for: Impact of a Quality Improvement Initiative to Reduce Medication-induced Kidney Injury
Source: Pediatr Qual Saf. 2026 Sep 4;11(5):e912. doi: 10.1097/pq9.0000000000000912 (PMC13544836; doi:10.1097/pq9.0000000000000912)

Nephrotoxic Medication Screening Process Flow Diagram

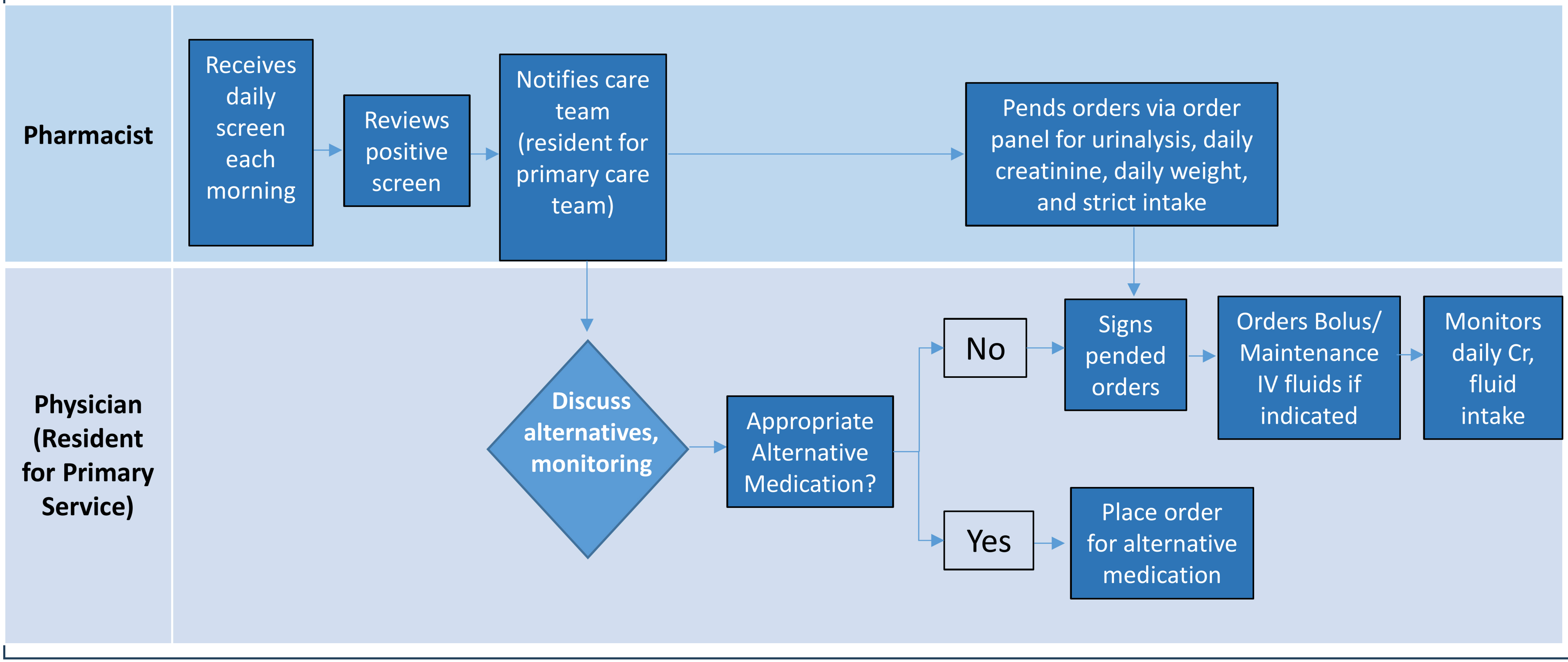

Supplement: Supplementary file 3 [file pqs-11-e912-s003.pdf]
